# Sensitivity and Selectivity on Aptamer-Based Assay: The Determination of Tetracycline Residue in Bovine Milk

**DOI:** 10.1100/2012/159456

**Published:** 2012-04-01

**Authors:** Sohee Jeong, Insook Rhee Paeng

**Affiliations:** Department of Chemistry, Seoul Women's University, Seoul 139-774, Republic of Korea

## Abstract

A competitive enzyme-linked aptamer assay (ELAA) to detect tetracycline in milk was performed by using two different aptamers individually; one is 76 mer-DNA aptamer and the other is 57 mer-RNA aptamer. The best optimum condition was obtained without monovalent ion, Na^+^ and also by adding no Mg^2+^ ion in the assay buffer, along with RT incubation. The optimized ELAA showed a good sensitivity (LOD of 2.10 × 10^−8^ M) with a wide dynamic range (3.16 × 10^−8^ M ~ 3.16 × 10^−4^ M). In addition, the average R.S.D. across all data points of the curve was less than 2.5% with good recoveries (~101.8%) from the milk media. Thus, this method provides a good tool to monitor tetracycline in milk from MRLs' point of view. However, this ELAA method was not superior to the ELISA method in terms of specificity. This paper describes that it does not always give better sensitivity and specificity in assays even though aptamers have several advantages over antibodies and have been known to be good binders for binding assays.

## 1. Introduction

Aptamer-based biosensing system has been widely investigated and developed to detect various proteins, small chemicals, and metabolites such as nucleic acids and carbohydrates in biological, environmental substance and food products [1–11]. Aptamers are functional oligonucleotides of single-stranded DNA or RNA which can recognize and bind to the specific target molecules with high affinity, specificity, and selectivity. As biomolecular recognizers, aptamers are used for diagnostic and therapeutic applications. Due to their small size, aptamers might be able to reduce steric hindrance and approach analytes more easily. Suitable aptamers can be selected by *in vitro* selection process called SELEX (systematic evolution of ligands by exponential enrichment) [2, 3] and generated in the short term compared with antibodies.

**Figure 1:**
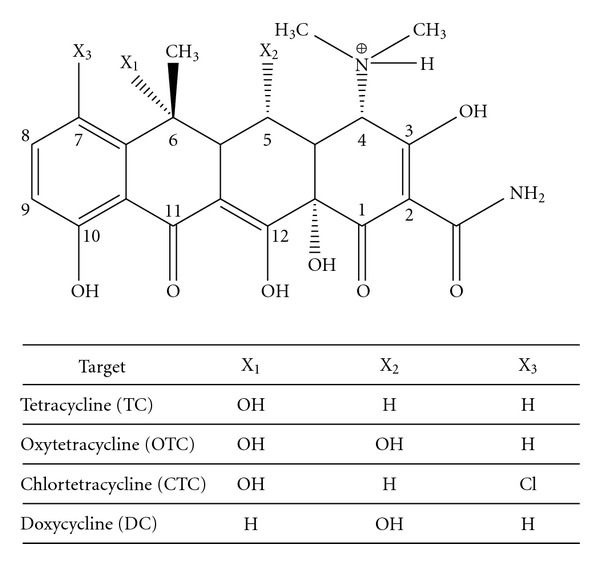
General structure of TCs.

Tetracyclines (TCs) are a group of broad-spectrum antibacterial agent. These have been widely used to treat bacterial infections in both humans and animals and to promote growth rate for animals. TCs are used in pure or mixed form with derivatives, oxytetracycline (OTC), chlortetracycline (CTC), and doxycycline (DC) (Figure 1) [12]. Therefore, food products from medicated animals such as meat [13], milk [14–16], fish [17, 18], and eggs [18] may contain TC residues and may cause serious health problems in people who take these food. According to the Codex MRLs (maximum residue levels) database, the MRLs of TCs are 100 *μ*g/kg in milk, 200 *μ*g/kg in muscle, 600 *μ*g/kg in liver, and 1200 *μ*g/kg in kidney (cattle).

Several methods have been reported for the detection of TCs using a variety of techniques, such as high-performance liquid chromatography (HPLC) and liquid chromatography-tandem mass spectrometry (LC-ESI-MS/MS) [19, 20]. These instrumental analyses provide simultaneous and accurate detection of TCs, including basic TC, OTC, DC, and 4-epitetracycline. However, these methods require expensive instruments and sample extraction and cleanup. There is a need for fast, easy to use, and cost-effective analytical methods. Indeed, well-established immunoassay techniques were developed to meet these requirements [21–23].

Recently, enzyme-linked immunosorbent assays were performed for determination of TC in food. These assays provide specificity, high sensitivity with less than 0.4 *μ*g/L of detection limit, and broad dynamic range that enable to detect target molecules [21–23]. Although the ability of antibodies to bind to their target is remarkable, there are some drawbacks, including a complicated process for antibody production and stability under elevated temperatures. By comparison, aptamers have several advantages. They are highly chemically stable over a wide temperature range, have reversible denaturation, and can be isolated by *in vitro* methods without immune response. Thus, aptamers are synthesized rapidly. DNA aptamer can be obtained in just a few days. Furthermore, they are easy to modify and label. A site-specific modification techniques are developed to attach various molecules such as biotin groups [9], thiol groups [24], and label molecules [8] at 3′ or 5′ end of aptamer without affecting the target binding site and can make one-to-one conjugate (aptamer : label) like gene-fusion technique [25–27].

TC has several binding aptamers, including 76 mer-DNA aptamer and 57 mer-RNA aptamer [12, 28]. 76 mer-DNA aptamer has high affinities for the basic TC backbone and has a dissociation constant (*K*_*d*_) of 63 nM [26, 27]. An electrochemical aptasensor has been studied utilizing this DNA aptamer [29, 30]. RNA aptamer can fold and form binding pocket to accommodate TC. It also binds to TCs tightly with a *K*_*d*_ of 770 pM, as determined by fluorimetry [28]. TC is a flat polycyclic molecule and has one aromatic ring. One face of the molecule makes ionic interaction with a part of RNA residues, while the other face associates with another part by multiple stacking or hydrophobic interactions [28, 31]. Several reports suggest that magnesium ions are also essential for TC binding, because RNA aptamer recognizes this metal ion that coordinates the TC [28, 31–33].

To stabilize the conformation of aptamer, monovalent cations such as Li^+^, Na^+^, and K^+^ have been used in aptamer assays as well as divalent ions. In some thrombin aptasensor studies, the effects of these cations were validated by addition of Na^+^ or K^+^ which can stabilize G-quadruplex structure and reduce nonspecific binding [34, 35]. However, Na^+^ or K^+^  is able to interact with negatively charged phosphate backbone of the aptamer and form weak complexes at higher concentration of cations [34]. Thus, they may lead to the conformational changes of binding site and cause aptamer's lower affinity to their target.

In this work, we establish a competitive enzyme-linked aptamer assay (ELAA) based on both DNA and RNA aptamers as recognition elements to determine residues of TC in milk samples. The biotinylated-aptamer was immobilized onto microplate via avidin-biotin interaction. Although the aptamers are known as binders with low nanomolar *K*_*d*_ values and exceptional recognition ability like antibodies, the specificity of aptamer has not been verified as well as that of antibodies. Also, we report that aptamers can measure the amount of TCs with maximum accuracy and provide the assay sensitivity (LOD) and % recovery rates in milk matrix.

## 2. Materials and Methods

### 2.1. Reagents

A single-stranded DNA aptamer was purchased from Genotech (Daejeon, Korea) and had the following sequences: 76 mer, 5′-CGT ACG GAA TTC GCT AGC CCC CCG GCA GGC CAC GGC TTG GGT TGG TCC CAC TGC GCG TGG ATC CGA GCT CCA CGT G-3′-biotin (Mw. (23746.2), mp (88.8°C)). The DNA aptamer was modified through 3′-end biotin modification using biotin-triethylene glycol (TEG).

An RNA aptamer was synthesized with a DMT- (dimethoxytrityl-) Biotin amidite at the 5′ end by ST Pharm (Seoul, Korea). The sequences are shown as the following: 57 mer, biotin-5′-GAG CCU AAA ACA UAC CAG AGA AAU CUG GAG AGG UGA AGA AUA CGA CCA CCU AGG CUC-3′ (Mw. (18869.7), mp (86.8°C)).

Hydrochlorides of TC, OTC, CTC, and DC were purchased from Sigma (St. Louis, Mo, USA). Neutravidin was obtained from Pierce (Rockford, Ill, USA). Bovine serum albumin (BSA), ovalbumin (OVA), trichloroacetic acid (TCA), Na_2_EDTA, 3,3′,5,5′-tetramethylbenzidine dihydrochloride (TMB), and phosphate-citrate buffer with sodium perborate were supplied from sigma (St. Louis, Mo, USA). Sodium bicarbonate, sodium carbonate, sodium phosphate monobasic, sodium phosphate dibasic, citric acid monohydrate, and sodium chloride were purchased from Duksan Pure Chemical (Ansan, Kyunggido, Korea). McIlvain buffer solution was prepared by dissolving 11.8 g of citric acid monohydrate, 13.72 g of Na_2_HPO_4_, and 33.62 g of Na_2_EDTA in one liter of distilled water [21]. All chemicals were of analytical grade, and triply deionized water (Milli-Q water purification system, Millipore, Billerica, Mass, USA) was used. All buffers were filtered using 0.20 *μ*m membrane filter system.

### 2.2. Instrumentation

ELAA was performed in Flat-bottomed polystyrene immuno plates (Nunc, Denmark). Enzyme activity was detected using E-max microtiter plate reader (Molecular Device Co., Sunnyvale, Calif, USA) at 450 nm. Microplates were washed using Multiwasher III (Tricontinent, Grass valley, Calif, USA). Centrifugation was performed in Supra22K from Hanil Sci. (Incheon, Korea).

### 2.3. Optimization of Assay Conditions

Prior to the DNA aptamer assay, the optimization was carried out by varying concentrations of aptamer and TC-HRP because the assay sensitivity is dependent on the concentrations of binder and competitor. The DNA aptamer was diluted to 0.025 *μ*g mL^−1^, 0.0188 *μ*g mL^−1^, and 0.0125 *μ*g mL^−1^. The TC-HRP conjugates were prepared in a concentration of 1 *μ*g mL^−1^ and 0.5 *μ*g mL^−1^. The optimization of amount of neutravidin was also performed in three different concentrations of 1 *μ*g mL^−1^, 0.5 *μ*g mL^−1^, and 0.1 *μ*g mL^−1^. The incubation temperature was examined by incubating DNA aptamer, standard TC solution, and TC-HRP at 4°C or RT, respectively. The assay was conducted with the aptamer with and without a thermal treatment before immobilization, and the results were compared. The thermal denaturation was performed by heating the aptamer at 95°C for 10 min and then cooling it rapidly in ice.

In the RNA aptamer assay, it was performed for optimization of operating conditions. The incubation temperature was also examined by incubating at 4°C or RT. Several buffers were compared as diluents for all materials, including 10 mM phosphate, PBS, and phosphate containing 2.5 mM and 5 mM Mg^2+^. To optimize the concentration of aptamer, the RNA aptamer solution was diluted as ranging from 0.02 *μ*g mL^−1^ to 0.1 *μ*g mL^−1^ in 10 mM phosphate buffer.

### 2.4. Enzyme-Linked Aptamer Assay for TC

Neutravidin was diluted to 1 *μ*g mL^−1^ in coating buffer (50 mM bicarbonate buffer, pH 9.6), and 100 *μ*L was added to wells. The plate was stored with constant shaking at 4°C for 16 h. To remove unbound neutravidin, wells were washed three times with 300 *μ*L well^−1^ of the wash buffer, PBS-T (10 mM PBS, pH 7.2, 0.05% Tween-20). The plate was then blocked with 300 *μ*L well^−1^ of blocking agents (3% bovine serum albumin (BSA) or 1% ovalbumin (OVA)) in 10 mM phosphate buffer for 30 min at RT, and unbound BSA was removed. Subsequently, 100 *μ*L of the biotinylated DNA aptamer (0.0188 *μ*g mL^−1^) or RNA aptamer (0.07 *μ*g mL^−1^) was added to each well, respectively, and binding was allowed for 1 h at RT. The unbound materials were removed by washing three times with the wash buffer (10 mM PBS-T). TC was diluted to 2 × 10^−3^ M–2 × 10^−12^ M in assay buffer (10 mM phosphate, pH 7.2). Each 50 *μ*L of various concentrations of TC solution was added in triplicate, and the plate was incubated at RT for 30 min. Then, 50 *μ*L TC-HRP (1 *μ*g mL^−1^) in assay buffer was added to the plate. An incubation step was accomplished for 30 min with constant shaking at RT. After washing, 100 *μ*L of TMB in substrate buffer (50 mM phosphate-citrate buffer, pH 5.0) was added and incubated for 10 min at RT. The color of solution changed from colorless to blue. The color reaction was stopped by adding 50 *μ*L of H_2_SO_4_ (2 M). Then, absorbance was read at 450 nm.

### 2.5. Cross-Reactivity Study

The cross-reactivity study was performed in the same way as the TC assay. OTC, CTC, and DC were used as cross-reactants. These are antibacterial agents with structure related to TC.

### 2.6. Pretreatment for Milk Assay

TC was spiked in milk. Milk contains protein, fat, carbohydrate, and cations such as Ca^2+^ and Mg^2+^ which can form chelation complexes with TC. To remove these inhabitants, 1 mL of milk sample in which TC spiked in advance was mixed with 4 mL of McIlvaine buffer contained 0.02 M EDTA (pH 4.0) and 0.4 mL of trichloroacetic acid was added to this mixture. Then, the milk sample was defatted and deproteinized by centrifugation at 4°C (5000 rpm, 20 min). 1 M NaOH was added dropwise to the supernatant to adjust to pH 7.2. The supernatant (5 × 10^−3^ M TC in milk sample) was prepared and serially diluted with treated milk sample (1 : 19 dilution, milk : phosphate buffer) in concentration ranging from 2 × 10^−3^ M to 2 × 10^−12^ M.

### 2.7. Recoveries of TC from Spiked Milk Samples

Free TC (9.60 mg L^−1^, 2.40 mg L^−1^, 0.48 mg L^−1^, 0.25 mg L^−1^, 0.10 mg L^−1^, and 0.05 mg L^−1^) was spiked in milk, in ten replicate samples.

## 3. Results and Discussion

The TC aptamer assay was performed by using two different aptamers individually; one is 76 mer-DNA aptamer and the other is 57 mer-RNA aptamer. The both aptamers has been known to have high affinities with a dissociation constant (*K*_*d*_) of 63 nM for DNA aptamer (76 mer) and 770 pM for RNA aptamer (57 mer). A competitive assay with sequential mode was adapted for both aptamer assays.

### 3.1. DNA Aptamer (76 Mer) Assay

**Figure 2:**
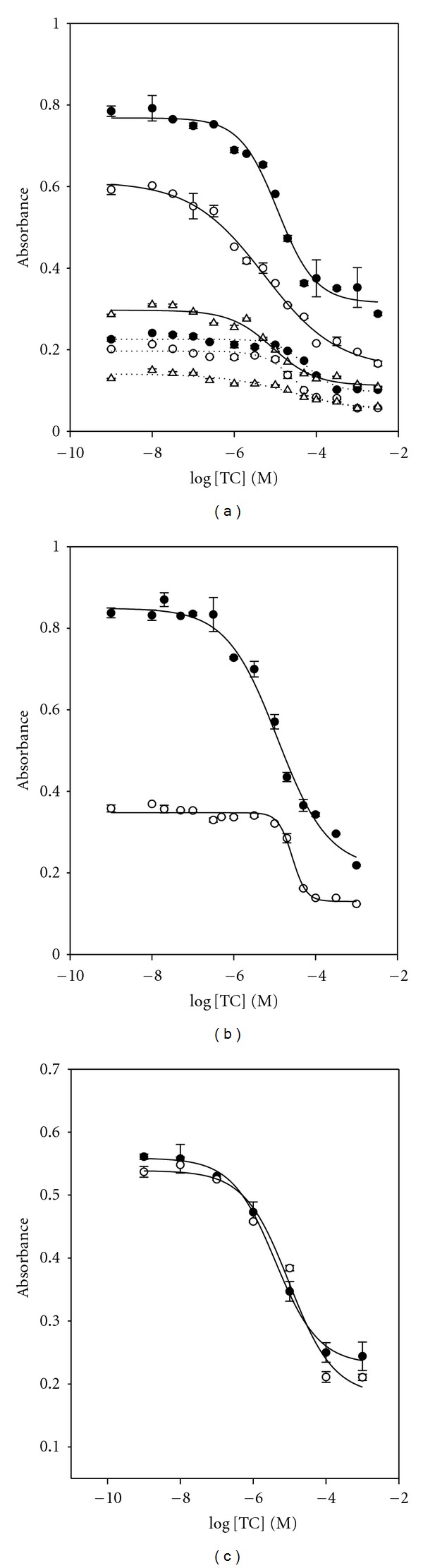
Optimization of TC assay conditions. (a) The concentration of DNA aptamer was (•) 0.0250 *μ*g mL^−1^, (∘) 0.0188 *μ*g mL^−1^, and (∆) 0.0125 *μ*g mL^−1^ and the concentration of TC-HRP conjugate was 1 *μ*g mL^−1^ in a solid line and 0.5 *μ*g mL^−1^ in a dotted line. (b) Effect of incubation temperature on ELAA performance: (•) room temperature and (∘) 4°C. (c) Influence of thermal denaturation of DNA aptamer: (•) 95°C, 10 min and (∘) no treatment.

The assay sensitivity for competitive assays depends on both the immobilized aptamer concentration on the solid surface and the limited concentration of the competitor, TC-HRP conjugate. Thus, the concentrations of aptamer (0.0250 *μ*g mL^−1^, 0.0188 *μ*g mL^−1^, and 0.0125 *μ*g mL^−1^) and the competitor, TC-HRP conjugate (1 *μ*g mL^−1^ and 0.5 *μ*g mL^−1^), are controlled to optimize the assay performance.  Figure 2(a) shows the binder dilution curve obtained by a matrix combination of both chosen reagents. The best detection limit was observed using a combination of 0.0188 *μ*g mL^−1^ of aptamer and 1 *μ*g mL^−1^ of TC-HRP. When 0.5 *μ*g mL^−1^ of the competitor, TC-HRP conjugate, was used with the same concentration of aptamer, the signal difference between the high and low doses of TC was too small (less than 0.1 Abs.) to be useful. This is shown in dotted line.

The effect of incubation temperature in the TC aptamer assay was examined by incubating aptamer at 4°C or at RT for 1 h each. As shown in Figure 2(b), the assay at 4°C shows the worse assay sensitivity with a small signal difference compared to that at RT. This result is different from the one obtained from dopamine RNA aptamer assay [9], in which the tertiary structure of DA aptamer is stable at lower temperature. Hereafter, experiments were performed at RT.

Additionally, an assay optimization was performed with the aptamer solution with and without a thermal treatment before its immobilization (Figure 2(c)). A thermal treatment of this biotinylated aptamer before the immobilization is applied to unfold the aptamer strand and also make the biotin label available for interaction with neutravidin on the solid support, plates. Thermal treatment involved the incubation of the aptamer solution at 90°C for 10 min, followed by rapid cooling in ice for 10 min to block the aptamer in its unfolded structure. The results were compared in terms of the TC binding. As shown in Figure 2(c), the aptamer denaturation did not show any improvement of the assay efficiency. This suggests that the TC aptamer holds the same folding structures even after the denaturation.

**Figure 3:**
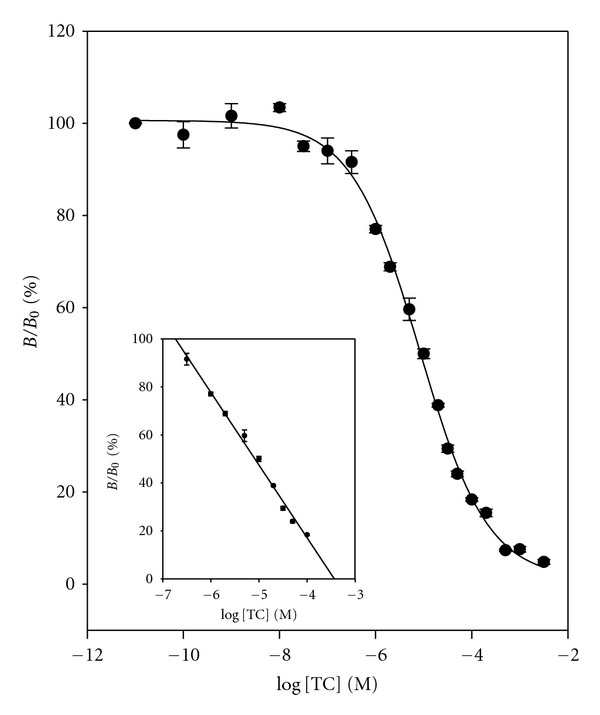
Dose-response curve and calibration curve (inset) for TC in buffer. %  *B*/*B*_0_ = −  30.3121 × log⁡  [TC] − 104.0775, *R* ^2^ = 0.9934. Data points are the average plus ± one standard deviation (*n* = 3).

Figure 3 shows the dose-response curve constructed with the optimized conditions using 0.0188 *μ*g mL^−1^ (7.5 × 10^−10^ M) of aptamer and 1.0 *μ*g mL^−1^ (1.5 × 10^−8^ M) of TC-HRP conjugate in assay buffer. The LOD of 3.27 × 10^−8^ M (15.7 *μ*g mL^−1^) and the dynamic range of from 1.00 × 10^−4^ M to 1.99 × 10^−7^ M were obtained. The LOD is defined as the concentration corresponding to 3 standard deviations below the mean from the blank. The average relative standard deviation (R.S.D.) across all data points of the curve was 1.6%. The calibration curve (inset) was constructed on a dynamic range, and least-squares regression [25] of these data gave the following relationship:

(1) $$\begin{array}{cc} \%\frac{B}{B_{0}} & =-30.3121\times \log\;\left[\text{TC}\right]\\ & \;-104.0775,\;\left(R^{2}=0.9934\right). \end{array}\;$$

**Figure 4:**
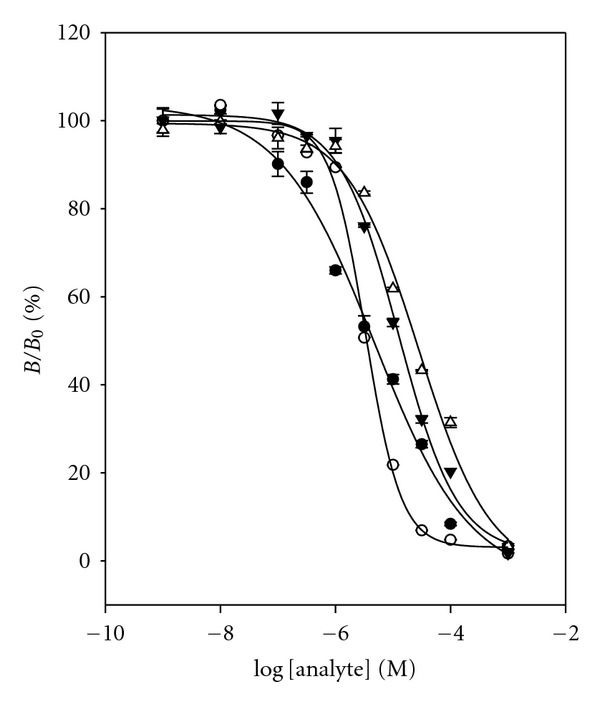
Cross-reactivity study with DNA aptamer for cross-reactants: (•) TC, (∘) OTC, (▾) CTC, and (*▵*) DC. Data points are means of triplicate measurements.

In an aptamer assay, the ability of an aptamer to yield a measurable response selectively for the target molecule is described as specificity [12]. The specificity of the aptamer was evaluated by cross-reactivity study using three structurally similar compounds, cross-reactants, such as OTC, CTC, and DC. Cross-reactivity studies were carried out by a competitive ELAA by adding various free cross-reactants at different concentrations to compete with competitor, TC-HRP, to bind with the aptamer coated on the surface. Their 50%  *B*/*B*_0_ values were estimated and then their percent cross-reactivity was calculated: % cross-reactivity = (concentration of TC giving 50%  *B*/*B*_0_)/(concentration of cross-reactant giving 50%  *B*/*B*_0_). *B*/*B*_0_ is the ratio of response *B*, to the maximum response when no analyte is present *B*_0_. The 50%  *B*/*B*_0_ value and cross-reactivity for each compound are given in Figure 4 and Table 1. These results demonstrated that OTC showed very high cross-reactivity (129.7%) and CTC and DC showed relatively high cross-reactivity with 32.4% and 17.5%, respectively. Table 1 lists the comparison of cross-reactivity, and TC DNA aptamer assay shows no superior specificity of the binder as compared to the TC antibody assay [21].

**Figure 5:**
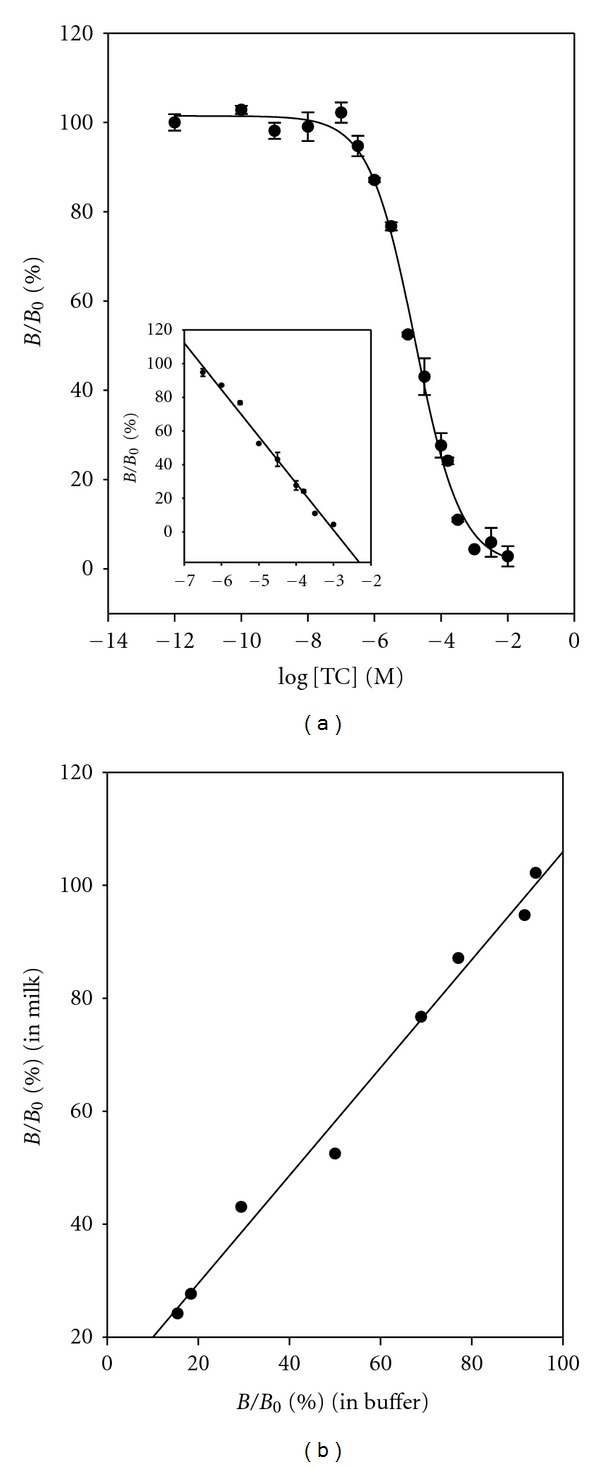
(a) Dose-response curve and calibration curve (inset) for TC in milk. %  *B*/*B*_0_ = −  27.7749 × log⁡  [TC] − 82.1902, *R* ^2^ = 0.9881. (b) Correlation diagram of *B*/*B*_0_ for TC both in buffer and in milk. %  *B*/*B*_0_  (in  milk) = 0.9550 × %  *B*/*B*_0_  (in  buffer) − 10.4243, *R* ^2^ = 0.9881.

Milk samples were obtained from local markets and stored in refrigerator before use. The protein and fat in milk sample have an inclination for forming chelating complexes with divalent ions, and they cause a matrix effect. To remove these interferences, the milk sample was treated with McIlvaine-EDTA buffer to block any interaction with TC and divalent cations. And then 20% trichloroacetic acid was used for denaturation of milk protein. The dose-response curve was performed using 0.0188 *μ*g mL^−1^ (7.5 × 10^−10^ M) of aptamer and 1.0 *μ*g mL^−1^ (1.5 × 10^−8^ M) of TC-HRP conjugate.  Figure 5(a) shows the dose-response curve and calibration curve for TC in milk. The LOD and the dynamic range were 9.52 × 10^−8^ M (45.7 *μ*g mL^−1^) and from 3.16 × 10^−4^ M to 3.16 × 10^−7^ M, respectively:

(2) $$\begin{array}{cc} \%\frac{B}{B_{0}} & =-\;27.7749\times \log\;\left[\text{TC}\right]\\ & \;-82.1902,\;\left(R^{2}=0.9881\right). \end{array}$$

Figure 5(b) shows the correlation diagram of the absorbance obtained both in buffer and in milk:

(3) $$\begin{array}{cc} \%\frac{B}{B_{0}}\;\left(\text{in}\;\text{milk}\right) & =0.9550\times \%\;\frac{B}{B_{0}}\;\left(\text{in}\;\text{buffer}\right)\\ & \;-10.4243,\;\left(R^{2}=0.9881\right). \end{array}$$

### 3.2. RNA Aptamer (57 Mer) Assay

Monovalent cations such as Li^+^, Na^+^, and K^+^ and divalent ion, Mg^2+^, have been used in aptamer assays to stabilize the conformation of aptamer. In the case of thrombin aptasensor studies, it is mentioned that an addition of Na^+^ or K^+^ can stabilize G-quadruplex structure and reduce nonspecific binding. On the other side, these monovalent ions can cause the conformational changes of aptamer binding site and produce a lower affinity to its target.

**Figure 6:**
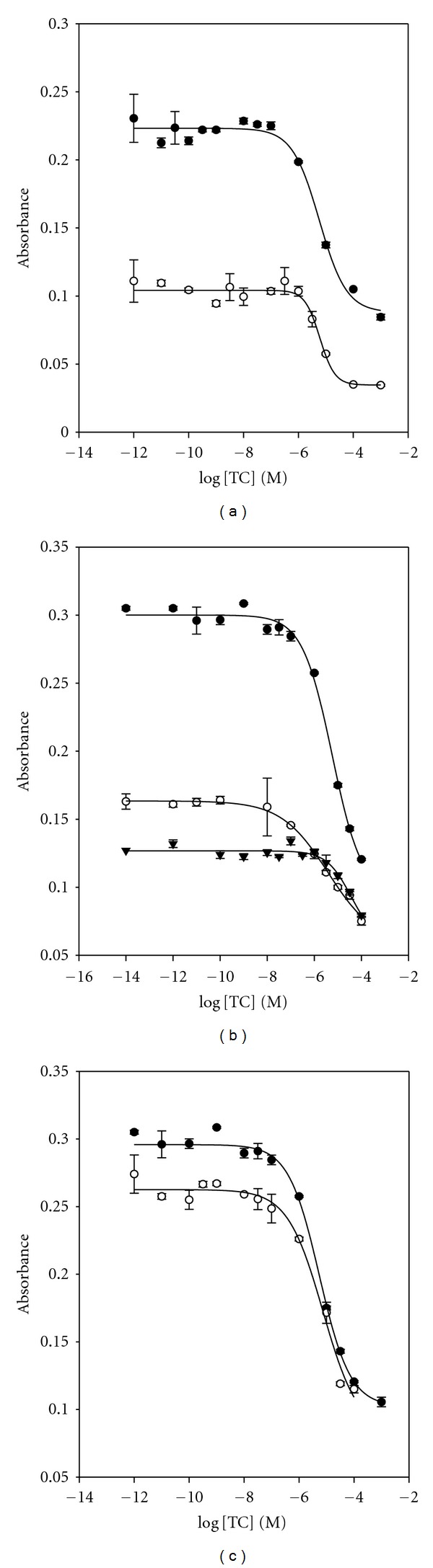
Optimization of assay conditions with RNA aptamer. (a) Influence of the addition of Na^+^ ion on assay buffer; (•) 10 mM phosphate buffer and (∘) PBS buffer (150 mM NaCl). (b) Effect of the addition of Mg^2+^ ion on assay buffer. The concentration of MgCl_2_ was (•) no addition, (∘) 2.5 mM, and (▾) 5 mM. (c) Optimization of temperature on the ELAA performance: (•) room temperature and (∘) 4°C.

In order to study whether monovalent and divalent cations affect the TC/RNA aptamer binding sensitivity, the assay buffer with and without monovalent and divalent cations were tried and the results compared.  Figure 6(a) shows the influence of the addition of Na^+^ ion in assay buffer. As shown in Figure 6(a), the maximum signal absorbance obtained with 10 mM phosphate buffer was compared with that obtained with 10 mM phosphate buffered saline, PBS buffer. In the case of absence of Na^+^ ion in the buffer solution, the dose-response curve showed that the signal gap was sufficiently large to detect the concentration of TC distinctly and with a lower detection limit than the one with Na^+^ cation.  Figure 6(b) shows the influence of the addition of Mg^2+^ ion in the assay buffer. Three different concentrations of Mg^2+^ ion were examined (0.0 mM, 2.5 mM, and 5.0 mM). As shown in Figure 6(b), the higher concentration of Mg^2+^ ion was used, the lower the signal gap was obtained. Several reports using UV-Vis spectroscopy [22] suggested that Mg^2+^ ion essentially binds to TC. This means that the TC forms the coordination complex with Mg^2+^ ion. Thus, Mg^2+^ ion can interfere with the binding reaction between TC and RNA aptamer. Incubation temperature was also studied for ELAA performance (RT and 4°C), and it showed no significant difference on both assay temperatures (Figure 6(c)).

**Figure 7:**
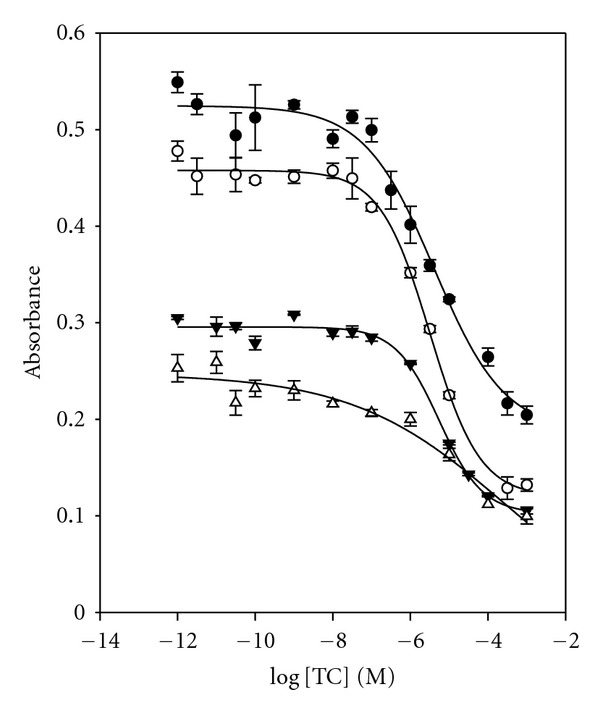
Optimization of the concentration of RNA aptamer: (•) 0.1 *μ*g mL^−1^, (∘) 0.07 *μ*g mL^−1^, (▾) 0.05 *μ*g mL^−1^, and (*▵*) 0.02 *μ*g mL^−1^ with the concentration of TC-HRP conjugate, 1 *μ*g mL^−1^.

The concentration of RNA aptamer was optimized for the assay.  Figure 7 shows the binder dilution curve obtained with four different RNA aptamer concentrations (0.1 *μ*g mL^−1^, 0.07 *μ*g mL^−1^, 0.05 *μ*g mL^−1^, and 0.02 *μ*g mL^−1^) and the concentration of TC-HRP conjugate (1.0 *μ*g mL^−1^). As shown in Figure 7, the best detection limit and signal gap were obtained using a combination of 0.07 *μ*g mL^−1^ of aptamer and 1.0 *μ*g mL^−1^ of TC-HRP.

**Figure 8:**
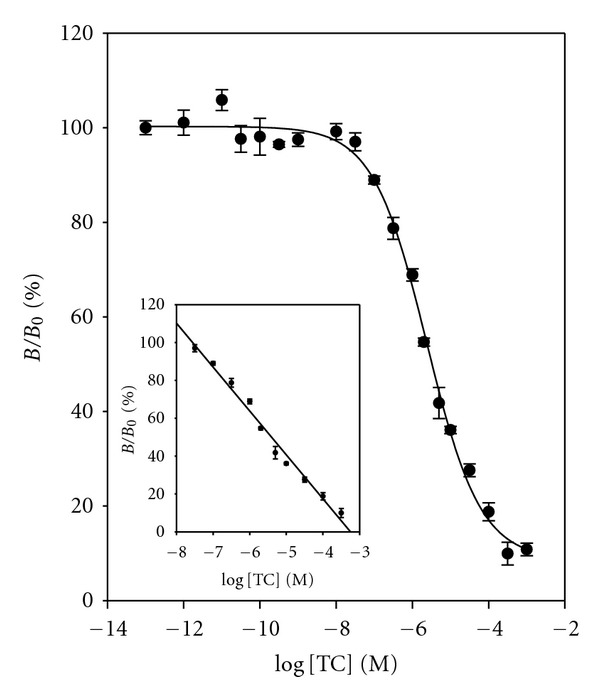
Dose-response curve and calibration curve (inset) for TC in buffer. %  *B*/*B*_0_ = −  23.1788 × log⁡  [TC] − 75.2471, *R* ^2^ = 0.9851. Data points are the average plus ± one standard deviation (*n* = 3).

Figure 8 shows the dose-response curve constructed with the optimized conditions using 0.07 *μ*g mL^−1^ (3.7 × 10^−9^ M) of RNA aptamer and 1.0 *μ*g mL^−1^ (1.5 × 10^−8^ M) of TC-HRP conjugate in the assay buffer. The LOD of 2.10 × 10^−8 ^M (10.1 *μ*g mL^−1^) and the dynamic range of from 3.16 × 10^−4^ M to 3.16 × 10^−8^ M were obtained. The calibration curve (inset) was constructed on a dynamic range, and these data gave the following relationship:

(4) $$\begin{array}{cc} \%\frac{B}{B_{0}} & =-23.1788\times \log\;\left[\text{TC}\right]\\ & \;-75.2471,\;\left(R^{2}=0.9851\right). \end{array}\;$$

**Figure 9:**
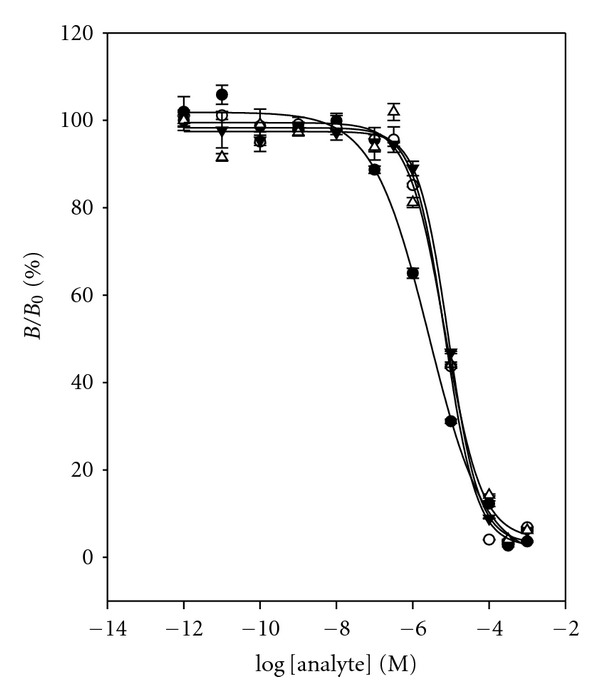
Cross-reactivity study with RNA aptamer for cross-reactants: (•) TC, (∘) OTC, (▾) CTC, and (*▵*) DC. Data points are means of triplicate measurements.

The specificity of the RNA aptamer was evaluated by cross-reactivity study. Cross-reactivity studies were carried out by a same method as previous DNA aptamer assay. The 50%  *B*/*B*_0_ value and cross-reactivity for each compound are given in Figure 9 and Table 1. These three cross-reactants demonstrated similar cross-reactivity (35.3%, 35.3%, and 30.4%) with relatively high value. It supports that the binding of TC to RNA aptamer is by hydrophobic or stacking interaction of the flat polycyclic aromatic ring in TC. Thus, RNA aptamer gives low specificity of the binder as compared to the TC antibody assay [21].

**Figure 10:**
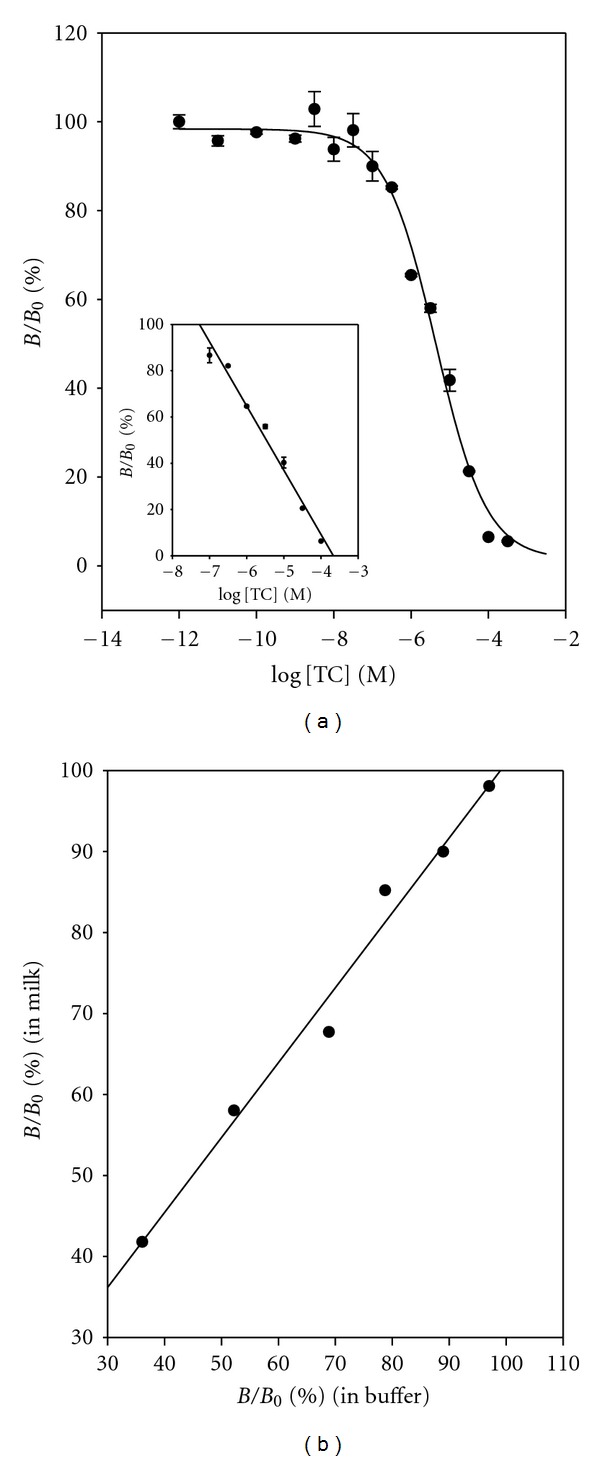
(a) Dose-response curve and calibration curve (inset) for TC in milk. %  *B*/*B*_0_ = −27.7674 × log [TC] − 101.8244, *R* ^2^ = 0.9823. (b) Correlation diagram of *B*/*B*_0_ for TC both in buffer and in milk. %  *B*/*B*_0_ (in milk) = 0.9252 ×  %  *B*/*B*_0_ (in buffer) + 8.4232, *R* ^2^ = 0.9837.

Figure 10(a) shows the dose-response curve and calibration curve for TC in milk. The LOD and the dynamic range were 3.51 × 10^−8^ M (16.8 *μ*g mL^−1^) and from 1.00 × 10^−4^ M to 1.00 × 10^−7^ M, respectively:

(5) $$\begin{array}{cc} \%\frac{B}{B_{0}} & =-27.7674\times \log\;\left[\text{TC}\right]\\ & \;-101.8244,\;\left(R^{2}=0.9823\right). \end{array}\;$$

Figure 10(b) shows the correlation diagram of the absorbance obtained both in buffer and in milk:

(6) $$\begin{array}{cc} \%\frac{B}{B_{0}}\;\left(\text{in}\;\text{milk}\right) & =0.9252\times \%\frac{\;B}{B_{0}}\;\left(\text{in}\;\text{buffer}\right)\\ & \;+\;8.4232,\;\left(R^{2}=0.9837\right). \end{array}$$

The recovery study was performed using commercial milk purchased from local market against DNA aptamer and RNA aptamer, respectively. Free TC was spiked in milk sample, and mean percent recoveries were analyzed. The recovery study was performed in ten replicates, and the results were quite satisfactory as seen in Table 2.

## 4. Conclusion

A competitive ELAA method to detect TC in milk was developed. The optimization of assay buffer including aptamer affects the sensitivity of the aptamer for the TC. Best results were obtained without monovalent ion, Na^+^, and also by adding no Mg^2+^ ion in the assay buffer, along with RT incubation. In the case of both monovalent and divalent ions, increased ionic strength resulted in a decreased sensitivity of the aptamer to TC. This may be explained with shielding effect of Na^+^ ion causing the conformational changes of the aptamer binding site, therefore producing a lower affinity to its target, TC. Additionally, aptamer recognizes the Mg^2+^ ions that essentially coordinate TC.

The optimized ELAA method showed a good sensitivity (LOD of 2.10 × 10^−8^ M) with a wide dynamic range (3.16 × 10^−8^ M~3.16 × 10^−4^ M). In addition, the average R.S.D. across all data points of the curve was less than 2.5% with good recoveries (~101.8%) from the milk media. Thus, this method provides a good tool to monitor TC in milk from MRLs' point of view. As shown in Tables 1 and 3, the analytical data from this ELAA method is compared with those reported previously by ELISA method [21, 22]. However, this ELAA method (DNA or RNA aptamer) was not superior to the ELISA method (antibody as a binder) in terms of specificity, detection limit (LOD), and dynamic range. It demonstrated that the affinity and specificity of these aptamer assays were not advantageous compared to the previous immunoassay that uses antibodies. This means that it does not always give better sensitivity and specificity in assays even though aptamers have several advantages over antibodies and have been known to be good binders for binding assays. This study is the first report that uses validated ELAA for the determination of TC in milk.

## Figures and Tables

**Table 1:** Cross-reactivity (%) of cross-reactants.

Reactants	Cross-reactivity (%)		
	DNA aptamer	RNA aptamer	Antibody [21]
Tetracycline (TC)	100	100	100
Oxytetracycline (OTC)	129.7	35.3	10
Chlortetracycline (CTC)	32.4	35.3	13.7
Doxycycline (DC)	17.5	30.4	—

**Table 2:** Recovery study for the determination of TC spiked in milk.

	Spiked conc. of TC (mg L^−1^)	Measured conc. (mg L^−1^)	Recovery (%)
DNA aptamer	9.60	9.119	95.0
	2.40	2.390	99.6
	0.48	0.463	96.5

RNA aptamer	0.25	0.246	105.9
	0.10	0.104	104.7
	0.05	0.545	109.1

**Table 3:** Analytical data for TC aptamer assay (ELAA) compared with TC antibody assay (ELISA).

Method	Matrix	LOD	Dynamic range	Linearity, *R* ^2^
ELAA (DNA)	Buffer	3.27 × 10^−8^ M	1.99 × 10^−7^ M~1.00 × 10^−4^ M	0.9934
	Milk	9.52 × 10^−8^ M	3.16 × 10^−7^ M~3.16 × 10^−4^ M	0.9881

ELAA (RNA)	Buffer	2.10 × 10^−8^ M	3.16 × 10^−8^ M~3.16 × 10^−4^ M	0.9850
	Milk	3.51 × 10^−8^ M	1.0 × 10^−7^ M~1.0 × 10^−4^ M	0.9823

ELISA	Buffer [21]	1.74 × 10^−10^ M	1.0 × 10^−9^ M~1.0 × 10^−6 ^M	0.9901
	Milk [21]	1.0 × 10^−10^ M	3.16 × 10^−10^ M~3.16 × 10^−7^ M	0.9862
	Honey [22]	3.98 × 10^−10^ M	3.16 × 10^−9^ M~3.16 × 10^−7^ M	0.9800
